# Supplementary material for: ZGSO Spinel Nanoparticles with Dual Emission of NIR Persistent Luminescence for Anti-Counterfeiting Applications
Source: Materials (Basel). 2023 Jan 28;16(3):1132. doi: 10.3390/ma16031132 (PMC9920861; doi:10.3390/ma16031132)
Supplement: Supplementary file 1 [file materials-16-01132-s001.zip › materials-2139081-supplementary.pdf]

materials

SUPPLEMENTARY INFORMATIONS

Article

ZGSO Spinel Nanoparticles with Multi-Emission of NIR Persistent Luminescence for Anticounterfeiting Applications

Guanyu Cai [1,2], Teresa Delgado [1], Cyrille Richard [2,*], Bruno Viana [1,*]

[1] Université PSL, Chimie ParisTech, CNRS, IRCP, 75005 Paris, France
[2] Université Paris Cité, CNRS, INSERM, UTCBS, Unité de Technologies Chimiques et Biologiques pour la Santé, Faculté de Pharmacie, 75006 Paris, France
* Corresponding authors: bruno.viana@chimieparistech.psl.eu; cyrille.richard@u-paris.fr

Table S1 : Names and formulas of the Ni^{2+} doped ZGSO samples

ZGSO-0:Ni^{2+} (ZGO:Ni^{2+})	$ZnGa_{1.99}Ni_{0.01}O_{3.995}$
ZGSO-1:Ni^{2+}	$Zn_{1.1}Ga_{1.79}Ni_{0.01}Sn_{0.1}O_{3.995}$
ZGSO-2:Ni^{2+}	$Zn_{1.2}Ga_{1.59}Ni_{0.01}Sn_{0.2}O_{3.995}$
ZGSO-3:Ni^{2+}	$Zn_{1.3}Ga_{1.39}Ni_{0.01}Sn_{0.3}O_{3.995}$
ZGSO-4:Ni^{2+}	$Zn_{1.4}Ga_{1.19}Ni_{0.01}Sn_{0.4}O_{3.995}$
ZGSO-5:Ni^{2+}	$Zn_{1.5}Ga_{0.09}Ni_{0.01}Sn_{0.5}O_{3.995}$
ZGSO-6:Ni^{2+}	$Zn_{1.6}Ga_{0.79}Ni_{0.01}Sn_{0.6}O_{3.995}$

Table S2 : Names and formulas of the co-doped ZGSO samples

ZGSO-3:Ni^{2+},Er^{3+}	$Zn_{1.3}Ga_{1.38}Ni_{0.01}Er_{0.01}Sn_{0.3}O_{3.995}$
ZGSO-3:Er^{3+},Cr^{3+}	$Zn_{1.3}Ga_{1.38}Er_{0.01}Cr_{0.01}Sn_{0.3}O_{4}$
ZGSO-3:Ni^{2+},Er^{3+},Cr^{3+}	$Zn_{1.3}Ga_{1.37}Ni_{0.01}Er_{0.01}$ $Cr_{0.01}Sn_{0.3}O_{3.995}$

Table S3: Name and formula of the Cr3+ doped ZGSO sample (no Ni^{2+} and no Er^{3+})

ZGSO-3:Cr^{3+}	$Zn_{1.3}Ga_{1.39}Cr_{0.01}Sn_{0.3}O_{4}$

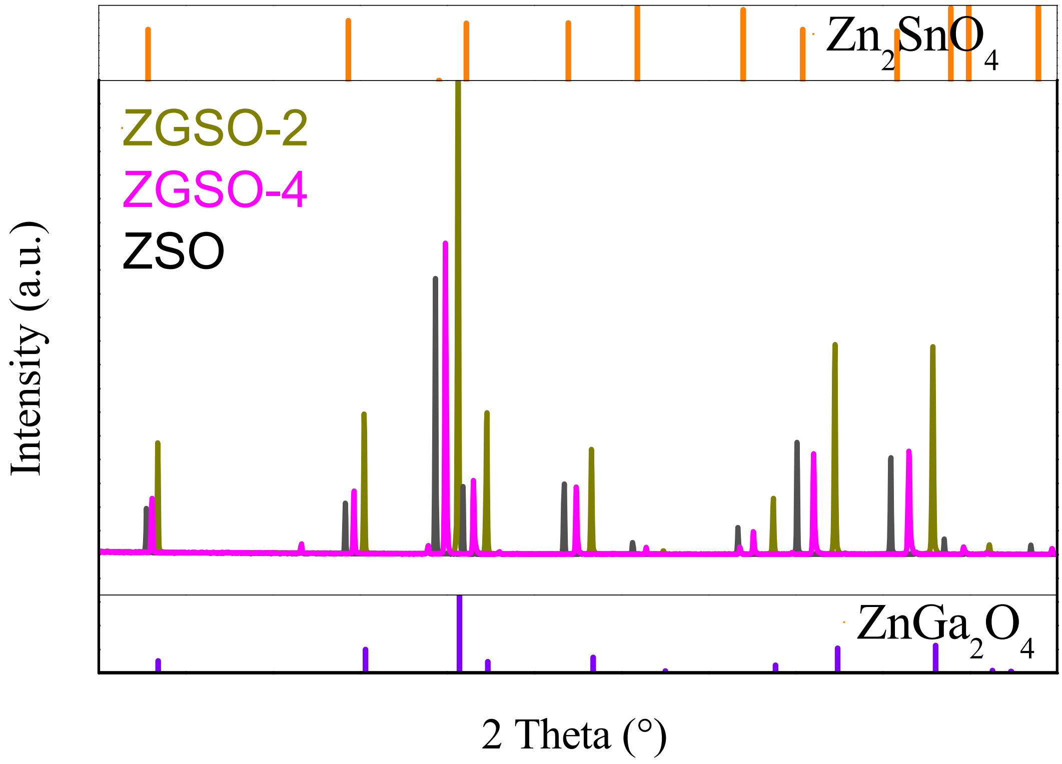

Figure S1 : XRD patterns of 0.5% Ni^{2+} doped ZGSO-2, ZGSO-4 and ZSO samples. With the increasing Sn^{4+} content, the normal spinel is kept until very high tin content but there is a shift observed in relation to the difference in cationic sizes (see main text).

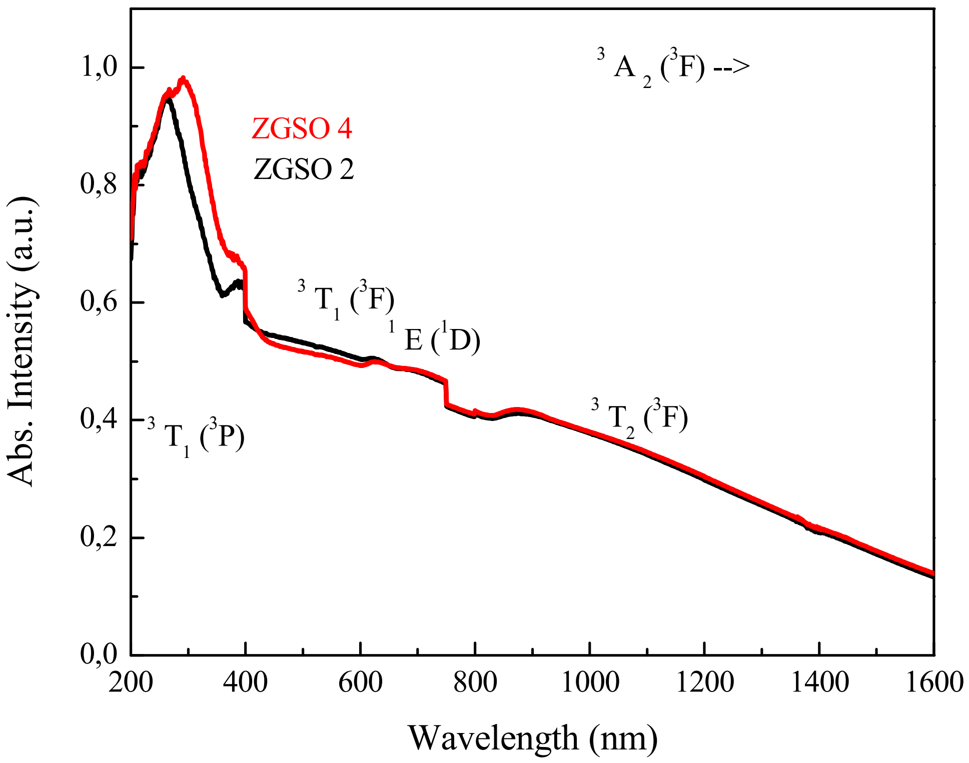

Figure S2 : Normalized absorption spectra of 0.5% Ni^{2+} doped ZGSO-2 and ZGSO-4 samples. At this very low doping doping content, the Ni^{2+} absorption variation is barely observed while the main effect is the tin variation of the host insuring energy bandgap variation (see main text).

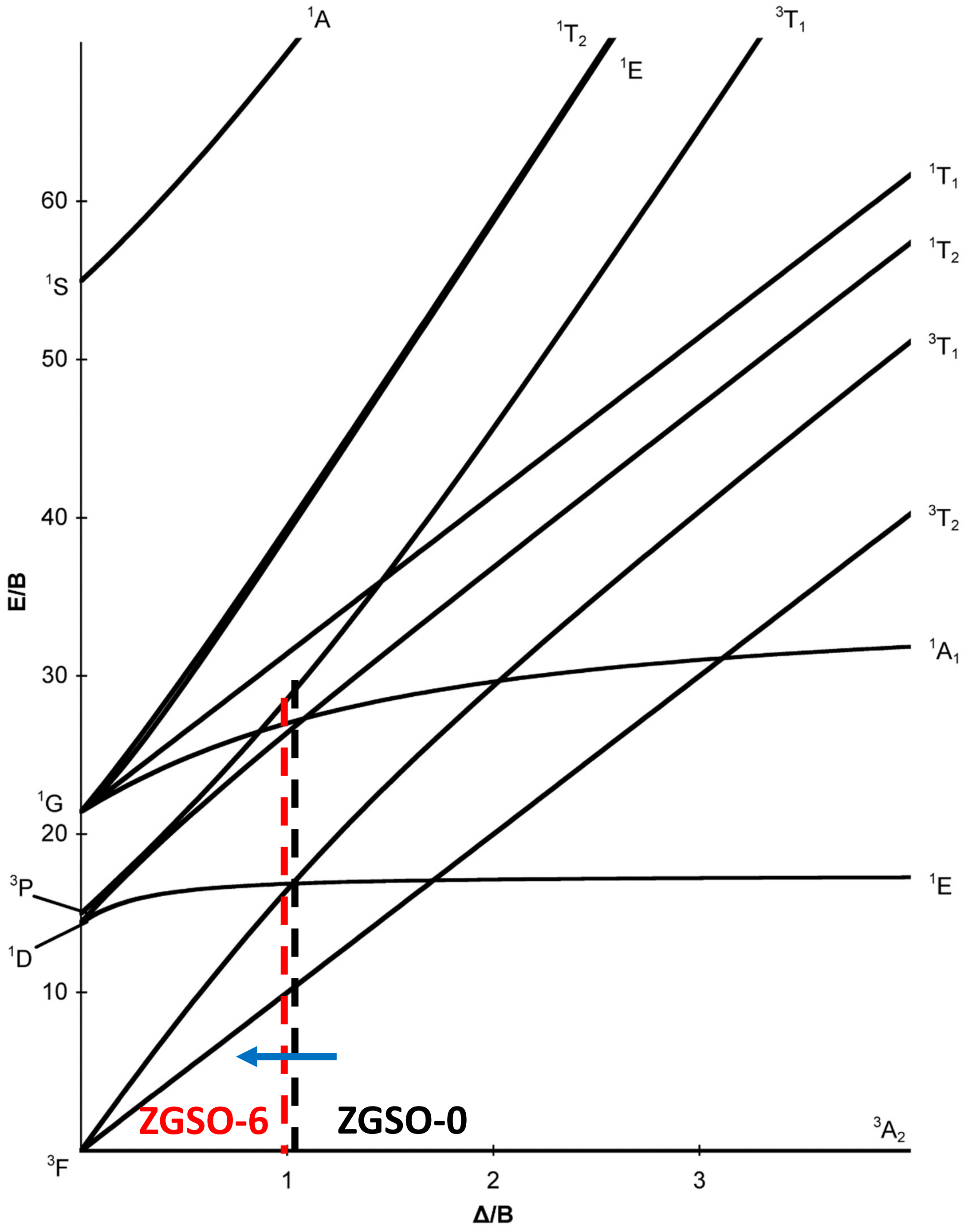

Figure S3 : Tanabe-Sugano diagram of Ni^{2+} (d^8 configuration) in the complex spinel samples of ZGSO-0 ($ZnGa_2O_4$:0.5%Ni^{2+}) and ZGSO-6 ($Zn_{1.6}Ga_{0.8}Sn_{0.6}O_4$:0.5%$Ni^{2+}$) corresponding to different Sn^{4+} concentrations.

(a)

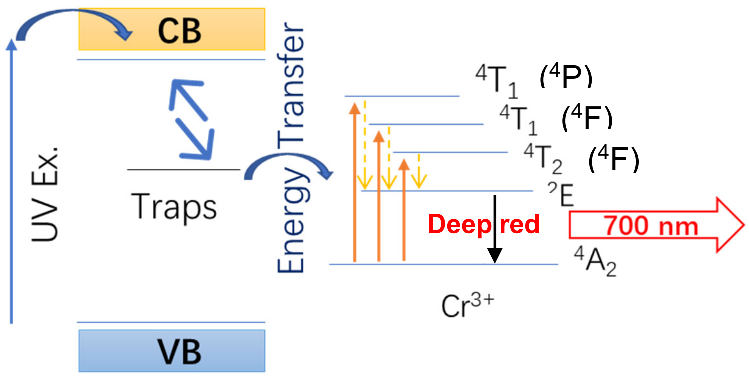

(b)

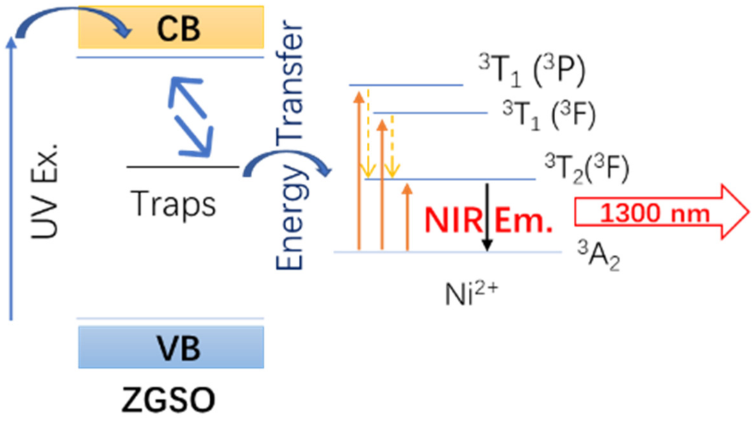

(c)

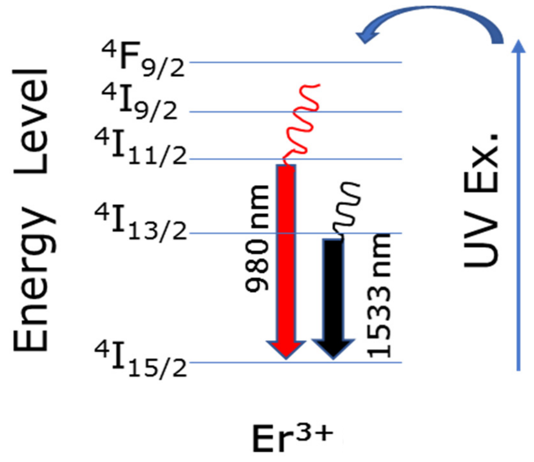

Figure S4 : Schematic diagrams of the absorption/emission bands; trapping/detrapping and energy transfer mechanisms leading to deep red emission of (a) ZGSO-3:Cr^{3+} NPs, and NIR emission of (b) ZGSO-3:Ni^{2+} NPs, and 4f-4f transition of (c) ZGSO-3:Er^{3+} NPs.

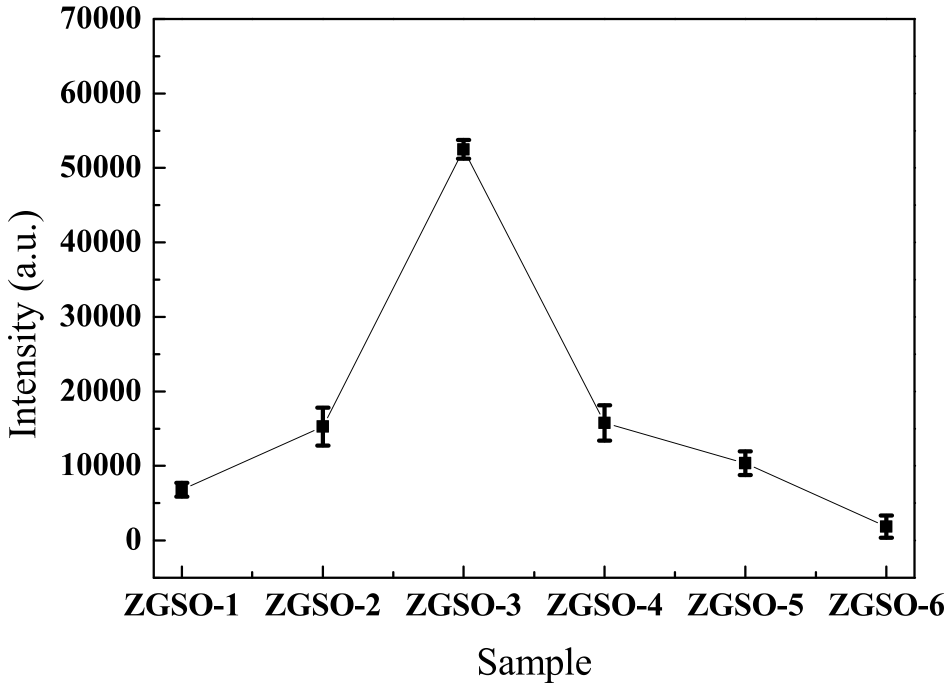

Figure S5 : Persistent luminescence ~~Persist. Lum.~~ intensity of Ni^{2+} doped ZGSO-0 –ZGSO-6 samples (detection 1 min after cutting off the 365 nm UV lamp excitation). Same quantity of powder and same excitation time is used for all samples. Detection with InGaAs camera.

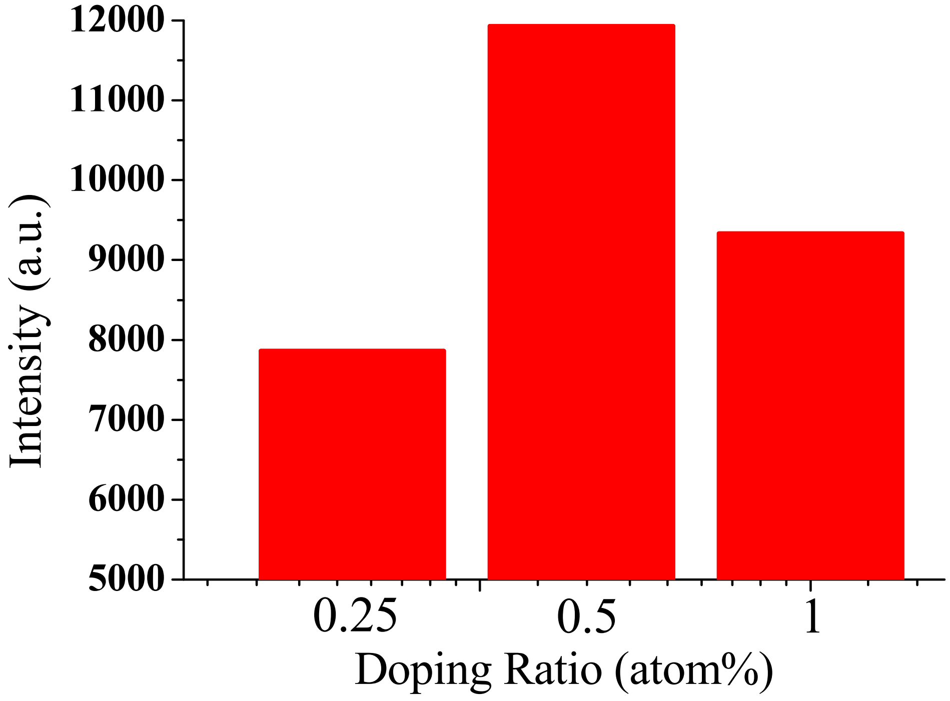

Figure S6 : Persistent luminescence ~~Persist. Lum.~~ intensity of the Ni^{2+} doped ZGSO-3 for three nominal different doping concentrations, namely 0.25%, 0.5% and 1%. (detection with the InGaAs camera, 1 min after cutting off the 365 nm UV lamp excitation).

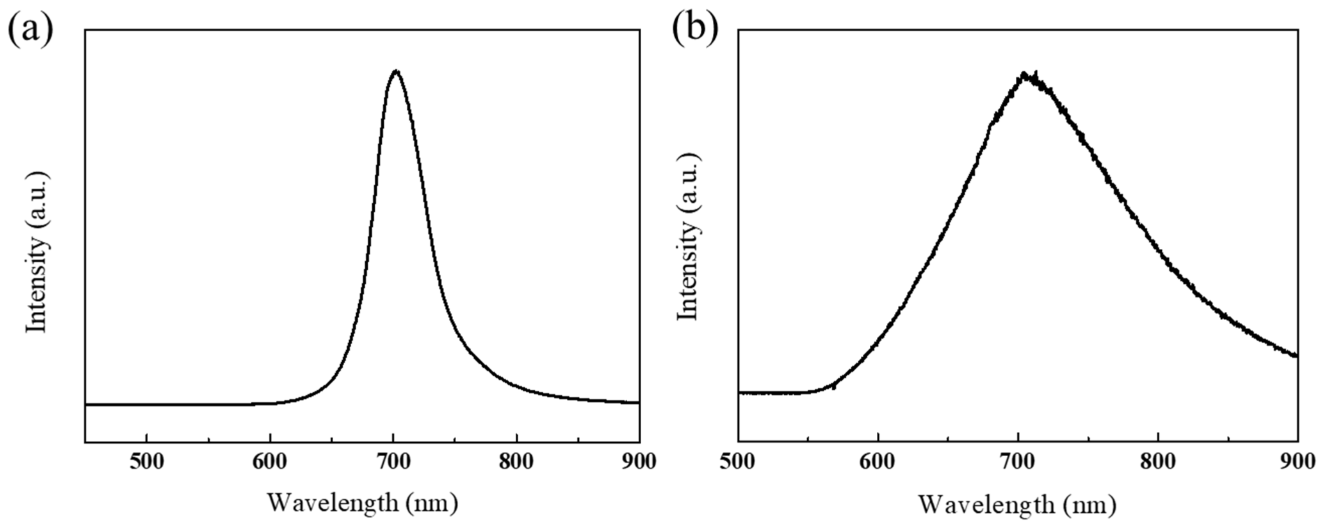

Figure S7 : Persistent luminescence ~~Persist. Lum~~. spectra of (a) ZGSO-3:Cr^{3+} and (b) ZGSO-3:Er^{3+}, Cr^{3+}, respectively. (detection with the Si-camera 1min after removal of UV lamp excitation (365nm, 5 min). No persistent emission from Er^{3+} is observed.

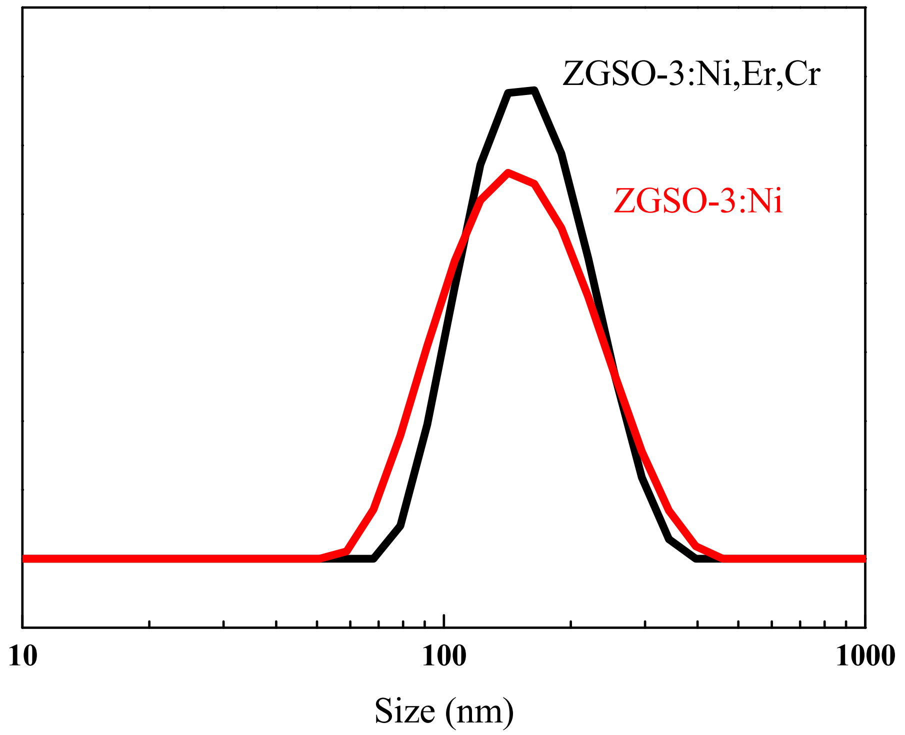

Figure S8 : Nanoparticles size measurements of ZGSO-3:Ni^{2+} and ZGSO-3:Ni^{2+},Er^{3+},Cr^{3+} samples obtained by DLS.

(a)

(b)

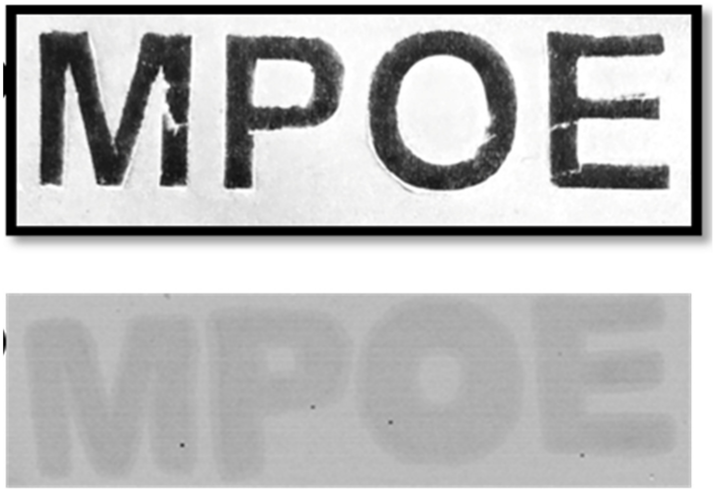

Figure S9 : (a) Image of the “MPOE” pattern templated by hand, and filled using ZGSO-3:Ni^{2+},Er^{3+},Cr^{3+} NPs, detacted by the *Si*-camera (b) PL image recorded from the “MPOE” pattern using ZGSO-3: Ni^{2+},Er^{3+},Cr^{3+} NPs under UV excitation, detected by *InGaAs*-based NIR camera with minimum contrast to avoid saturation.

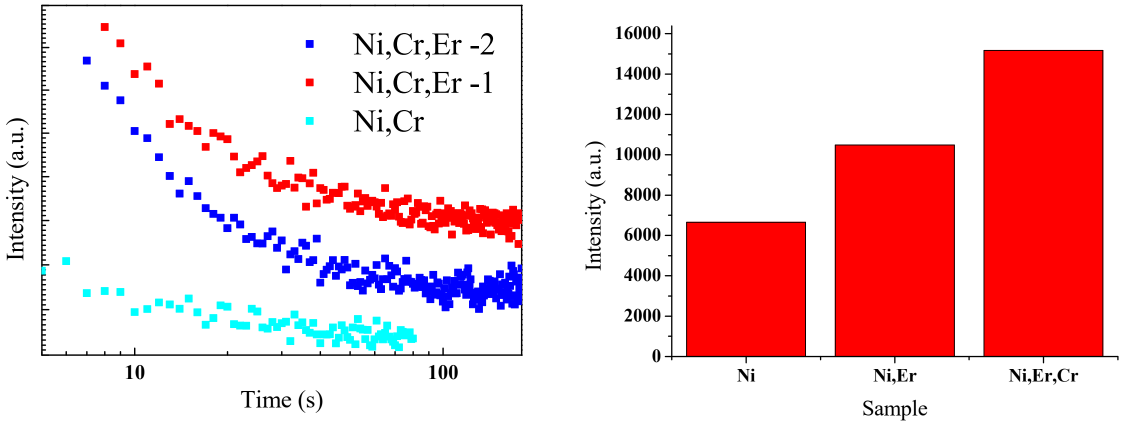

Figure S10. a) Decay of persistent luminescence of the three samples (ZGSO:Ni ; ZGSO:Ni,Er ; ZGSO:Ni,Er,Cr) measured with the InGaAs camera. b) Intensity of NIR persistent luminescence at 1300 nm of three samples (ZGSO:Ni ; ZGSO:Ni,Er ; ZGSO:Ni,Er,Cr). The detection of the persistent luminescence of all the samples started 1 min after cutting off the 365 nm UV lamp excitation. Same experimental conditions for the 3 samples.

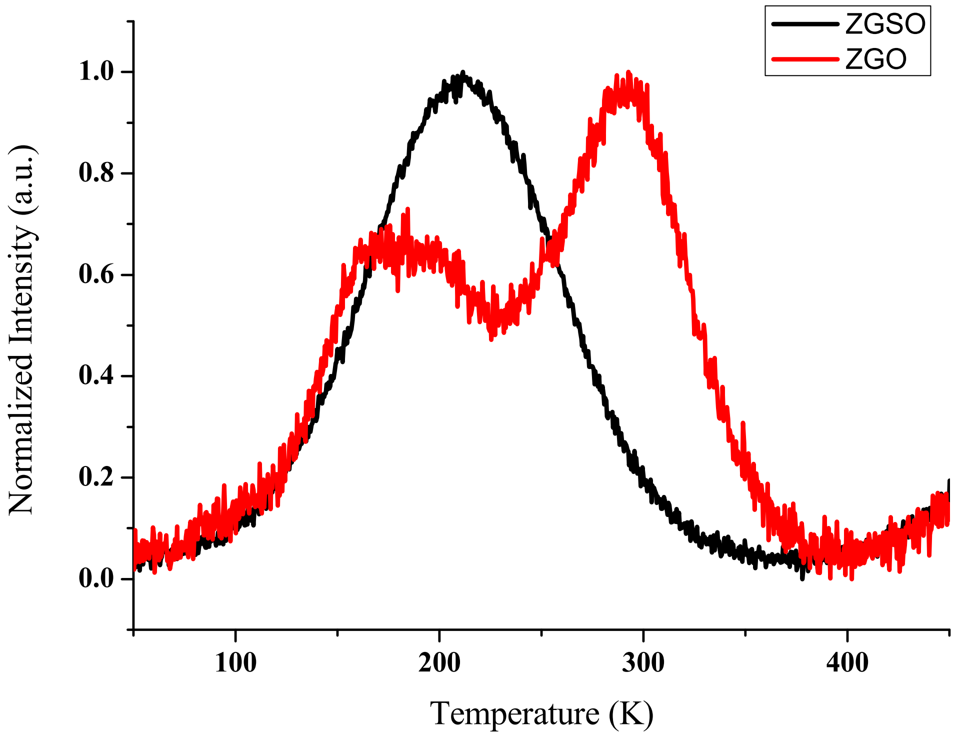

Figure S11: Normalized thermoluminescence glow curves of ZGSO:Ni and ZGO:Ni as obtained after 365 nm UV excitation during 5 minutes at 14 K (the emission wavelength is ~ 1300 nm.).
